# Reduction of Gastrointestinal Bleeding in Patients With Heyde Syndrome Undergoing Transcatheter Aortic Valve Implantation

**DOI:** 10.1161/CIRCINTERVENTIONS.122.011848

**Published:** 2022-07-05

**Authors:** Lia C.M.J. Goltstein, Maxim J.P. Rooijakkers, Natasha C.C. Görtjes, Reinier P. Akkermans, Erwin S. Zegers, Ron Pisters, Marleen H. van Wely, Kees van der Wulp, Joost P.H. Drenth, Erwin J.M. van Geenen, Niels van Royen

**Affiliations:** Department of Gastroenterology and Hepatology (L.C.M.J.G., N.C.C.G., J.P.H.D., E.J.M.v.G.), Radboud University Medical Center, Nijmegen, the Netherlands.; Department of Cardiology (M.J.P.R., M.H.v.W., K.v.d.W., N.v.R.), Radboud University Medical Center, Nijmegen, the Netherlands.; Scientific Institute for Quality of Healthcare (IQ Healthcare) (R.P.A.), Radboud University Medical Center, Nijmegen, the Netherlands.; Department of Cardiology, Canisius Wilhelmina Ziekenhuis, Nijmegen, the Netherlands (E.S.Z.).; Department of Cardiology, Rijnstate, Arnhem, the Netherlands (R.P.).

**Keywords:** angiodysplasia, aortic valve stenosis, capillaries, hemorrhage, prevalence, transcatheter aortic valve replacement

## Abstract

**Background:**

Heyde syndrome is the co-occurrence of aortic stenosis and gastrointestinal bleeding secondary to angiodysplasias. Surgical aortic valve replacement effectively reduces bleeding, but the effects of transcatheter aortic valve implantation (TAVI) are largely unknown. This study aimed to describe the reduction of gastrointestinal bleeding in patients with Heyde syndrome after TAVI and to identify the factors associated with rebleeding.

**Methods:**

We enrolled patients with Heyde syndrome from a prospective TAVI registry. Gastrointestinal bleeding episodes were assessed by the Bleeding Academic Research Consortium classification, and cumulative incidence functions were used to calculate cessation rates. Factors potentially associated with rebleeding were analyzed using logistic regression. Differences between Heyde and non-Heyde patients were assessed through a case-cohort study.

**Results:**

Between December 2008 and June 2020, 1111 patients underwent TAVI. There were 70 patients with Heyde syndrome (6.3%). In the first year following TAVI, gastrointestinal bleeding ceased in 46 of 70 patients (62% [95% CI, 50%–74%]). Bleeding episodes decreased from 3.2 (95% CI, 2.5–4.2) to 1.6 ([95% CI, 1.2–2.2] *P*=0.001) and hemoglobin levels increased from 10.3 (95% CI, 10.0–10.8) to 11.3 (95% CI, 10.8–11.6) g/dL (*P*=0.007). Between 1 and 5 years after TAVI (35 [interquartile range, 21–51] months), 53 of 62 patients (83% [95% CI, 72%–92%]) no longer experienced gastrointestinal bleeding. Paravalvular leakage (≥mild) was associated with rebleeding risk (odds ratio, 3.65 [95% CI, 1.36–9.80]; *P*=0.010). Periprocedural bleeding was more common in Heyde than in control patients (adjusted odds ratio, 2.55 [95% CI, 1.37–4.73]; *P*=0.003).

**Conclusions:**

Patients with Heyde syndrome are at increased risk for periprocedural bleeding. Post-TAVI, gastrointestinal bleeding disappears in the majority of patients. Paravalvular leakage may curtail these clinical benefits.

**What is Known**

- Heyde syndrome consists of aortic stenosis, acquired von Willebrand disease, and angiodysplasias and is an important differential diagnosis of gastrointestinal bleeding.
- Surgical aortic valve replacement effectively reduces gastrointestinal bleeding, but the effects of transcatheter aortic valve implantation on the reduction of gastrointestinal bleeding are less known.

**What the Study Adds**

- Gastrointestinal bleeding ceased in 62% of patients in the first year following transcatheter aortic valve implantation, which was maintained up to 5 years after the procedure.
- Paravalvular leakage was the only factor associated with gastrointestinal rebleeding episodes after transcatheter aortic valve implantation.
- Periprocedural bleeding was more frequent in patients with Heyde syndrome than in controls and was primarily of gastrointestinal origin.

Heyde syndrome is an important differential diagnosis of gastrointestinal bleeding in patients with aortic stenosis (AS).^1^ Bleeding originates from angiodysplasias, which are vascular malformations that consist of thin-walled, dilated arterial or venous capillaries in the gastrointestinal mucosa.^2^ These frail vessels easily rupture, which causes bleeding.^3^ Angiodysplasias have been described in 2% to 10% of patients with AS, but the true prevalence is likely higher as small bowel assessment is not part of routine clinical workup in these patients.^4–6^

Patients with Heyde syndrome have an increased bleeding risk, as high shear stress around the stenotic valve causes inordinate proteolysis of von Willebrand factor multimers.^7^ This acquired form of von Willebrand disease combined with angiodysplasias results in a high requirement of blood transfusions, reduced quality of life, increased morbidity and mortality, and steep hospital costs.^7, 8^ The grade of AS is associated with the loss of von Willebrand factor multimers, which stresses the indication for valve replacement in these patients.^9^ Surgical aortic valve replacement (SAVR) has been described to reduce gastrointestinal bleeding episodes in up to 92% of Heyde patients.^1,10^

Transcatheter aortic valve implantation (TAVI) has become the standard treatment for patients with AS who have an increased surgical risk and is also gaining territory over SAVR in patients at intermediate and low surgical risk.^11^ Both gastrointestinal and periprocedural bleeding contribute greatly to morbidity and mortality after TAVI.^12^ A recent study showed that gastrointestinal bleeding ceased completely in 40% of Heyde patients in the first year following valve implantation.^4^ There are multiple outstanding questions. The extent and durability of the reduction in bleeding episodes and the concomitant rise in hemoglobin levels are unknown.^4,13^ Residual paravalvular leakage (PVL) after TAVI might contribute to rebleeding, but the effect size is unclear.^4,7^ Lastly, the risk of periprocedural bleeding in Heyde patients is unknown. In the present study, we assessed the effect of TAVI on gastrointestinal bleeding in Heyde patients from a large prospective registry up to 5 years after the procedure and determined which factors are associated with rebleeding. In a case-cohort study, we evaluated which baseline characteristics are associated with Heyde syndrome and whether Heyde patients are at an increased risk for periprocedural bleeding and adverse outcomes after TAVI.

## Methods

The data that support the findings of this study are available from the corresponding author upon reasonable request. This study was performed in accordance with the Strengthening the Reporting of Observational Studies in Epidemiology guidelines for reporting observational studies.^14^ All patients undergoing TAVI in the Radboud University Medical Center are part of an ongoing prospective registry that collects clinical and laboratory data. Informed consent for this registry was deemed unnecessary according to the Dutch Act on Medical Research Involving Human Subjects. To also collect data from the referring centers, the present study was approved by the Institutional Review Board of the Radboud University Medical Center, Nijmegen, the Netherlands (Commissie Mensgebonden Onderzoek Arnhem-Nijmegen; submission 2020-6697), and the 5 referring centers gave local approval (Canisius-Wilhelmina Ziekenhuis, Nijmegen; Rijnstate, Arnhem; Maasziekenhuis Pantein, Boxmeer; Slingeland Ziekenhuis, Doetinchem; and VieCuri Medisch Centrum, Venlo). Informed consent was obtained from all patients who were not deceased when the data were obtained.

### Study Enrollment and Data Collection

Heyde patients were identified through a manual and automated review of electronic medical records from all patients undergoing TAVI in one academic center between December 2008 and June 2020. All patients were part of a strict protocol collecting predefined data points, including regular cardiac ultrasounds and laboratory investigations.^15^ Additional details have been described in a previous publication.^16^ Heyde syndrome was defined as having gastrointestinal bleeding episodes (overt or occult) either (1) due to endoscopically diagnosed angiodysplasias or (2) due to a high suspicion of angiodysplasias (Table S1) but without complete endoscopic evaluation (consisting of gastroscopy, colonoscopy, and small bowel assessment through either capsule endoscopy or enteroscopy). We excluded patients diagnosed with an alternative explanation for gastrointestinal bleeding (eg, ulcer or cancer).

Baseline characteristics (including medical history and echocardiographic features) and data on gastrointestinal bleeding episodes the year before and after TAVI were extracted retrospectively from the electronic medical records. Hemoglobin levels one to several days before TAVI and ≈1 year after TAVI were noted. If multiple levels were determined, the levels closest to TAVI and 1 year after TAVI were used. Hemoglobin levels determined <6 months after TAVI were omitted.

To perform a case-cohort study, we established a random cohort from the TAVI registry in a 1:3 ratio. Stratification was used based on the year each Heyde patient underwent TAVI, as the indication for this procedure changed over time.^11^ Control patients who had iron deficiency anemia before TAVI with an explanation for gastrointestinal bleeding other than angiodysplasias but without complete endoscopic evaluation were excluded because the concomitant presence of angiodysplasias could not be ruled out (Table S1).

### Primary and Secondary Outcomes

Our primary outcome measure was the proportion of Heyde patients with complete cessation of gastrointestinal bleeding episodes between 72 hours and 1 year after TAVI. All bleeding episodes were scored in accordance with the Bleeding Academic Research Consortium classification, for which types 2 to 5 were used. We expanded the definition of bleeding to also capture occult bleeding (Table S2). Of note, a positive fecal occult blood test and endoscopic evaluation without application of treatment modalities were not scored as bleeding episodes.

Secondary outcome measures included the mean difference in hemoglobin levels, the reduction in the number of bleeding episodes, and corresponding health care utilization (consisting of blood transfusions, intravenous iron infusions, emergency department visits, hospital admissions, and endoscopic procedures) the year before and after TAVI (including the periprocedural period of 72 hours), the cessation of gastrointestinal bleeding up to 5 years after TAVI, and characteristics associated with rebleeding after TAVI. Comorbidities and medication previously related to rebleeding episodes of angiodysplasias (eg, chronic kidney disease, multiple angiodysplastic lesions, and use of antithrombotic therapy) and characteristics related to (acquired) von Willebrand disease (eg, blood group O, severe valvular disorders other than aortic valve stenosis, and PVL) were investigated.^9,17–20^

Sensitivity analyses including only patients with endoscopically confirmed Heyde syndrome were performed, as well as analyses including only patients who received a next-generation valve.

Heyde patients were compared with control patients to investigate baseline characteristics associated with Heyde syndrome, whether Heyde syndrome is associated with an increased proportion of periprocedural bleeding episodes (defined as bleeding episodes ≤72 hours after TAVI), and 1-year all-cause mortality.

### Statistical Analyses

Baseline characteristics and periprocedural bleeding episodes of Heyde patients and control patients are presented as mean with SD in case of normally distributed data or as median with interquartile range (IQR) in case of non-normally distributed data. Binary and categorical variables are presented as frequencies and percentages. Differences between Heyde and control patients in baseline characteristics and outcomes were analyzed with the independent *t* test or the Mann-Whitney *U* test when comparing continuous variables.^21^ Categorical variables were analyzed with the Pearson χ^2^ test or the Fisher exact test where appropriate.^22^

Time until gastrointestinal bleeding up to 1 year and 5 years after TAVI was assessed with cumulative incidence functions, using mortality as a competing event since this precludes bleeding. Cessation rates (1 minus the incidence rates) were displayed with 95% CIs.

The mean differences in bleeding episodes, hemoglobin levels, and health care utilization were compared with linear regression in case of continuous variables and negative binomial regression in case of count variables. We accounted for reduced follow-up durations due to mortality using the logistically transformed follow-up time after TAVI as an offset term in the model. Outcomes of the negative binomial regression analyses were expressed as geometric mean differences and corresponding incidence rate ratios.

Logistic regression was used to adjust for possible differences in baseline characteristics between Heyde patients and controls. The variable of interest (periprocedural bleeding or 1-year mortality) and baseline characteristics that differed between both groups were selected for the multivariate model. Logistic regression was also used to look for a potential association between baseline characteristics and procedure-related factors on the risk of gastrointestinal rebleeding after TAVI within the Heyde patients. We selected variables by using backward selection. Univariate analyses were performed, and variables with *P*<0.2 were selected for the multivariate model. Selected variables were entered at the same time into a model, and the least significant variable was removed from the model until only variables with a *P* of ≤0.05 were left. Outcomes were expressed as adjusted odds ratios (ORs) and 95% CIs.

A 2-tailed *P* of ≤0.05 was considered significant in all statistical analyses. Most statistical analyses and sampling of control patients were performed with the SPSS statistical software package, version 25.0 (IBM Corp, Armonk, NY). Cumulative incidence functions were performed with R, version 4.0.0 (R Foundation for Statistical Computing, Vienna, Austria).

## Results

**Figure 1.**
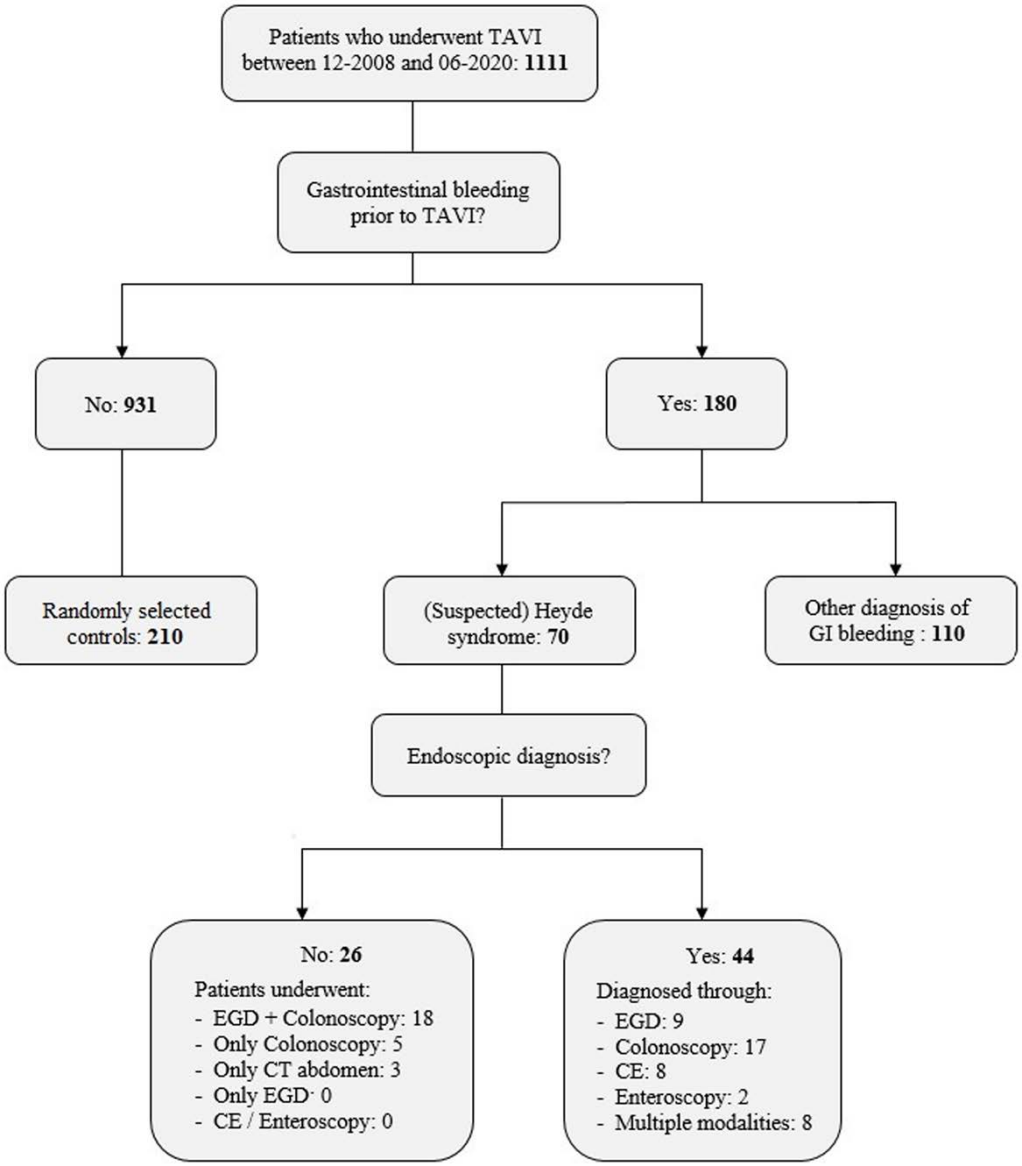
**Flowchart of the study.** The graph shows how patients with Heyde syndrome were selected from the Transcatheter Aortic Valve Implantation (TAVI) registry. Control patients were randomly selected in a 1:3 ratio. CE indicates capsule endoscopy; CT, computed tomography; EGD, esophagogastroduodenoscopy; and GI, gastrointestinal.

A total of 1111 patients underwent a TAVI procedure between December 2008 and June 2020 (Figure 1). We identified 70 Heyde patients (6.3%), concerning 44 (63%) patients with endoscopically confirmed angiodysplasias and 26 patients with confirmed gastrointestinal bleeding episodes but incomplete endoscopic evaluation (ie, suspected Heyde syndrome). One hundred and ten patients experienced gastrointestinal bleeding attributed to a cause other than angiodysplasias.

### Baseline Characteristics

Patients with Heyde syndrome had a median age of 78 years (IQR, 73–83), with an equal number of men (n=35) and women (n=35). Comorbidities were common among patients, including diabetes (39%) and chronic kidney disease (33%; Table 1). Eighty-seven percent of patients used antithrombotic therapy before TAVI, primarily single antiplatelet or oral anticoagulant therapy (60%) and dual antiplatelet therapy (24%). Most patients with endoscopically diagnosed angiodysplasias had multiple lesions (64%). Angiodysplasias were mainly located in the colon (24%) and small bowel (21%). Sixty-four (91%) patients had gastrointestinal bleeding episodes in the year before TAVI. Most patients received a self-expanding valve (90%), consisting of the Medtronic CoreValve (25/63), the Medtronic Evolut R (23/63), and the Abbott Portico (15/63). Seven (10%) patients received a balloon-expandable Edwards SAPIEN 3 valve. The Medtronic Evolut R, Abbott Portico, and Edwards SAPIEN 3 are next-generation valves.

**Table 1.**
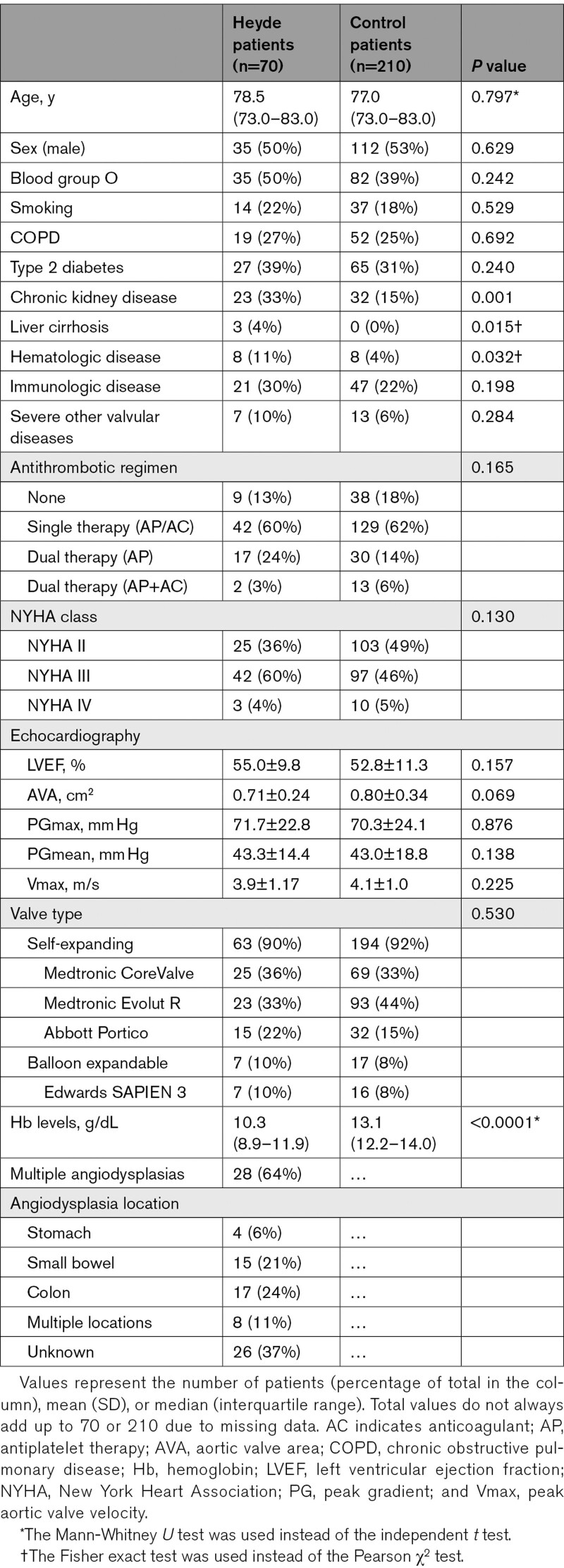
Baseline Characteristics of Heyde Patients and Control Patients

### Gastrointestinal Bleeding in Heyde Patients After TAVI

**Figure 2.**
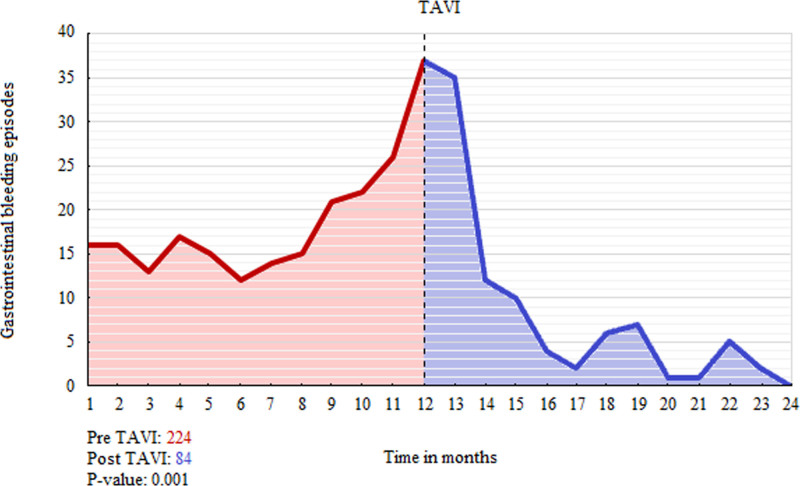
**Difference in gastrointestinal bleeding episodes before and after transcatheter aortic valve implantation (TAVI).** Graph of the difference in the number of gastrointestinal bleeding episodes per patient in the year before and after TAVI. Bleeding episodes within the periprocedural period (≤72 h) are included.

Of the 70 patients with Heyde syndrome, 46 (62% [95% CI, 50%–74%]) no longer experienced gastrointestinal bleeding episodes 72 hours to 1 year after TAVI. Thirty-seven (51% [95% CI, 40%–64%]) patients no longer experienced any gastrointestinal bleeding episodes, while 9 (11%) patients only experienced bleeding episodes during the periprocedural period (≤72 hours after TAVI). Twenty-four (38%) patients continued to experience gastrointestinal bleeding, of which 11 (16%) had an increase in bleeding episodes in the year after TAVI. Three of these patients did not experience gastrointestinal bleeding episodes in the year before TAVI (Figure 2).

**Figure 3.**
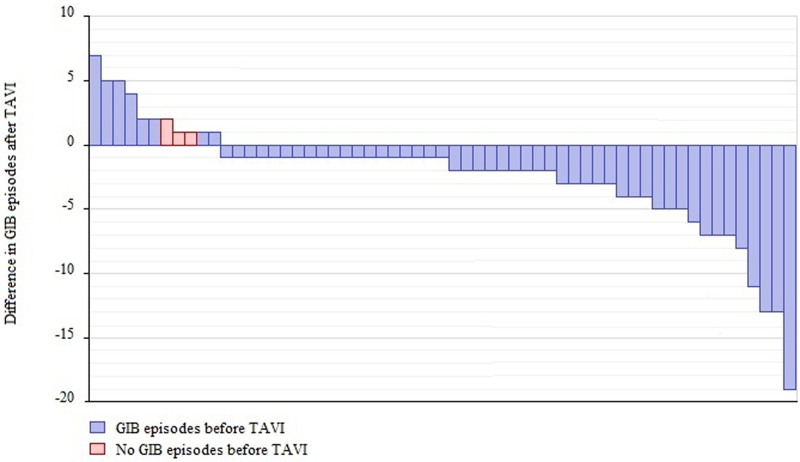
**Gastrointestinal bleeding (GIB) episodes before and after transcatheter aortic valve implantation (TAVI).** Graph of the total number of GIB episodes per month in the year before and after TAVI.

The mean number of gastrointestinal bleeding episodes significantly decreased in the year following TAVI compared with the year before from 3.2 (95% CI, 2.5–4.2) to 1.6 ([95% CI, 1.2–2.2] *P*=0.001). The severity of gastrointestinal bleeding also decreased (Table 2). Recurrent gastrointestinal bleeding episodes were mainly seen directly or in the first months after TAVI, with a steady decline during follow-up (Figure 3; Figure S1). The reduction in bleeding episodes was accompanied by a significant increase in hemoglobin levels following TAVI (median, 375 [IQR, 361–409] days); from 10.3 (95% CI, 10.0–10.8) to 11.3 (95% CI, 10.8–11.6) g/dL (*P*=0.007). The number of blood transfusions and iron infusions significantly decreased in the year after TAVI (4.8 versus 2.8, *P*=0.050 and 1.0 versus 0.5, *P*=0.033, respectively). The mean number of endoscopic procedures, emergency department presentations, and days admitted to the hospital due to gastrointestinal bleeding decreased numerically, but these differences were not significant (Table 2). Sensitivity analyses including only patients with endoscopically confirmed Heyde syndrome (44/70) also showed a significant decrease in bleeding episodes (3.6–2.0; *P*=0.021) and a significant increase in hemoglobin levels (10.3–11.1 g/dL; *P*=0.041) after TAVI (Table S3). Sensitivity analyses including only patients who received a next-generation valve (45/70) showed a significant decrease in bleeding episodes (2.5–1.1; *P*=0.007) and an increase in hemoglobin levels (11.3–12.1 g/dL; *P*=0.084) after TAVI (Table S4).

**Table 2.**
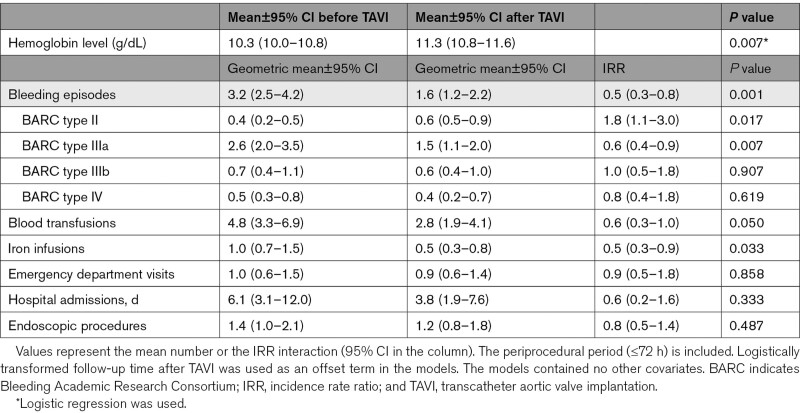
Difference in the Number of Gastrointestinal Bleeding Episodes and Corresponding Health Care Utilization 1 Year Before and After TAVI in Heyde Patients (n=70)

In addition, we examined the occurrence of gastrointestinal bleeding episodes up to 5 years after TAVI. Patients with Heyde syndrome had a median follow-up of 35 months (IQR, 21–51). Fifty-three of 62 (83% [95% CI, 72%–92%]) patients who had available data >1 year after TAVI did not experience gastrointestinal bleeding episodes 1 to 5 years after TAVI (Figure S2).

### Characteristics Associated With Rebleeding After TAVI

More than trace PVL (≥mild) was independently associated with a higher risk of rebleeding episodes than none or trace PVL (OR, 3.65 [95% CI, 1.36–9.80]; *P*=0.010). Comorbidities, number and location of angiodysplasias, and antithrombotic therapy after TAVI were not significantly associated with rebleeding after TAVI (Tables 3 and 4).

**Table 3.**
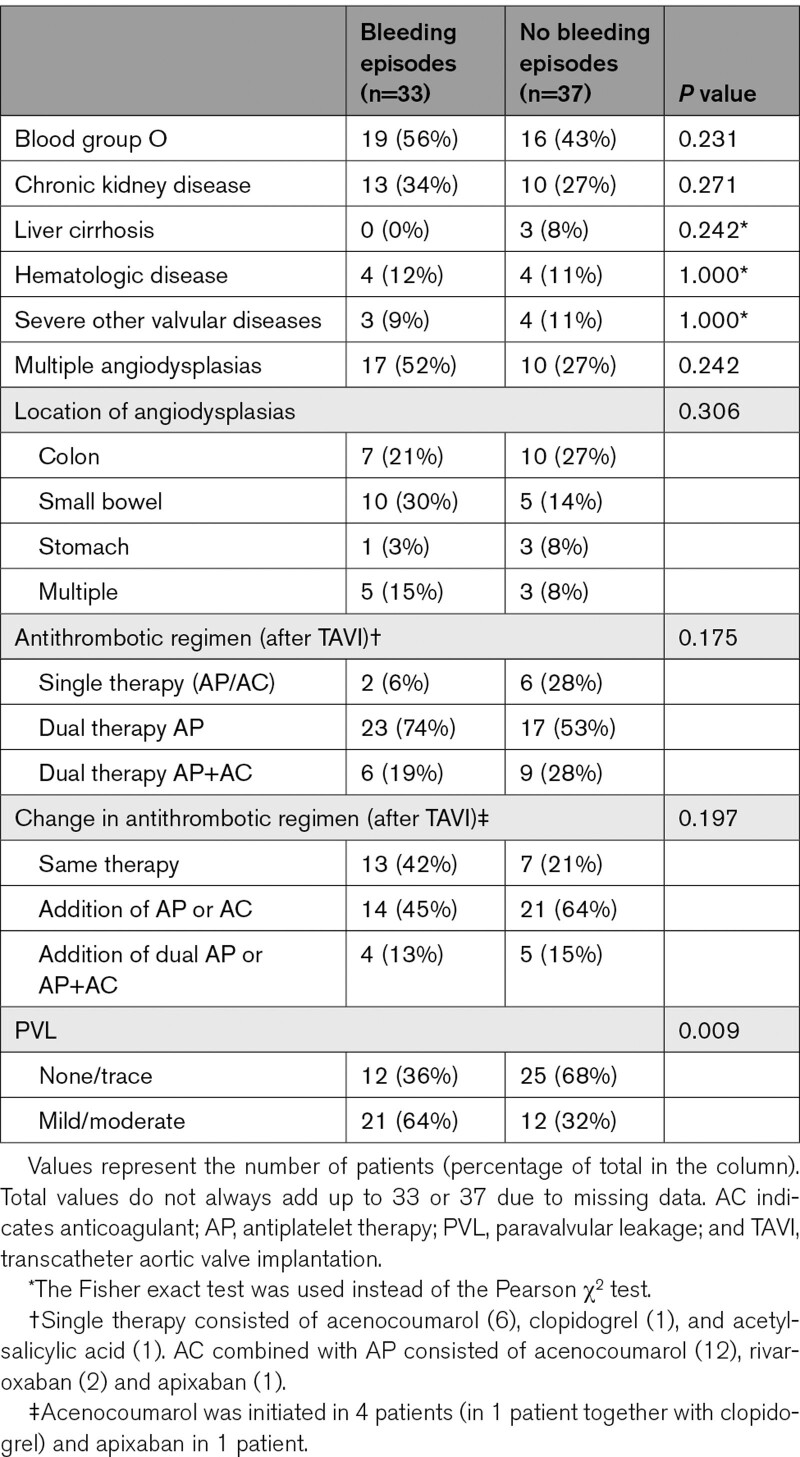
Baseline Characteristics of Heyde Patients With and Without Rebleeding After TAVI

**Table 4.**
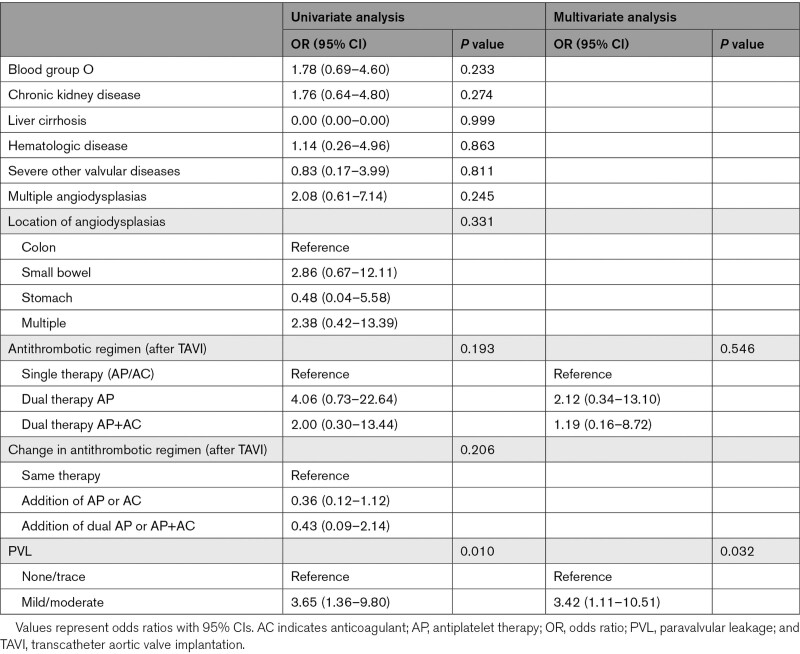
Factors Associated With Rebleeding After TAVI

### Characteristics Associated With Heyde Syndrome and Procedural Outcomes

In Heyde syndrome patients, chronic kidney disease (33% versus 15%; *P*=0.001), hematologic disorders (11% versus 3%; *P*=0.015), and liver cirrhosis (4% versus 0%; *P*=0.032) were more prevalent than in control patients (Table 1). The aortic valve area was smaller in Heyde syndrome patients than in control patients, but this difference was not significant (0.71±0.24 versus 0.80±0.34 cm^2^; *P*=0.069).

**Figure 4.**
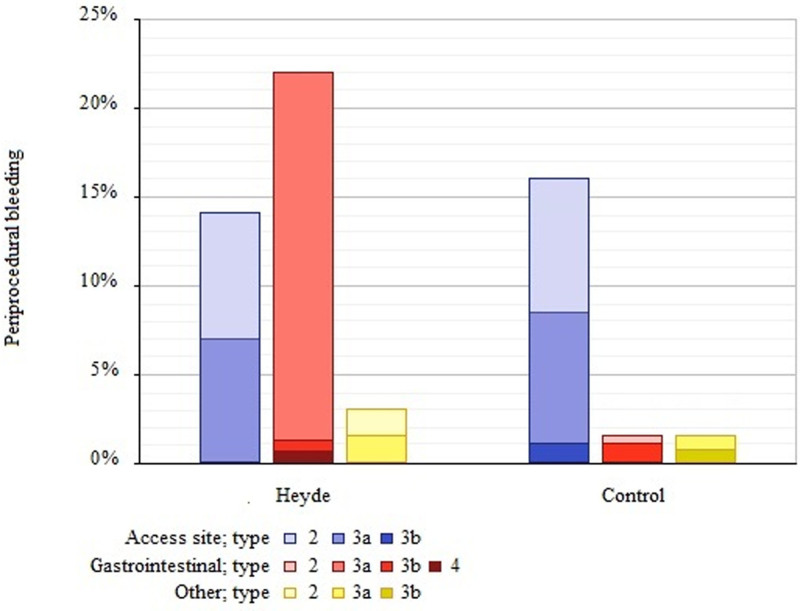
**Periprocedural bleeding episodes.** Graph illustrates the percentage of patients with periprocedural bleeding episodes ≤72 h after transcatheter aortic valve implantation. The type of bleeding (access site–related, gastrointestinal, and other non-access site–related bleeding) and the severity in accordance with the adjusted Bleeding Academic Research Consortium classification are reported.

Periprocedural bleeding episodes (≤72 hours after TAVI) occurred more often in Heyde syndrome patients compared with control patients (39% versus 19%). This yielded an adjusted OR of 2.55 ([95% CI, 1.37–4.73] *P*=0.003). In Heyde patients, most periprocedural bleeding episodes were gastrointestinal (15/27 [56%]), followed by access site–related bleeding (10/27 [37%]) and non-access site–related bleeding other than gastrointestinal (2/27 [7%]). In control patients, periprocedural bleeding episodes were access site related in the majority of cases (34/40 [85%]), followed by an equal number of gastrointestinal and other non-access site–related bleeding episodes (3/40 [8%]). The distribution and severity of periprocedural bleeding episodes among Heyde syndrome and control patients are displayed in Figure 4.

One-year mortality after TAVI occurred in 8 (11%) patients with Heyde syndrome. All of these patients died between 89 and 326 days after TAVI (median, 160 days). There were no periprocedural deaths, and no deaths were bleeding related. One-year mortality occurred in 17 (8%) control patients (adjusted OR, 1.52 [95% CI, 0.62–3.76]; *P*=0.363).

## Discussion

TAVI led to the complete cessation of gastrointestinal bleeding in 62% of Heyde syndrome patients in the first year after the procedure. This was associated with a large reduction in gastrointestinal bleeding episodes and a concomitant rise in hemoglobin levels. The beneficial effect was durable, and 83% had no gastrointestinal bleeding during a 5-year follow-up (35 [IQR, 21–51] months). Postprocedural PVL increased the risk of gastrointestinal rebleeding. The periprocedural bleeding rate was higher in patients with Heyde syndrome than in control patients, but 1-year mortality rates did not differ.

Aortic valve replacement has long been recognized as a favorable treatment option in patients with Heyde syndrome, as it resolves acquired von Willebrand disease.^1^ Not only does this dispel the bleeding diathesis of patients but also halts angiogenesis secondary to a lack of the von Willebrand factor.^23^ Both would result in a durable reduction of gastrointestinal bleeding episodes. Two studies analyzed patients with angiodysplasias who underwent SAVR. They included 16 and 57 patients and reported complete cessation in 92% and 79%, respectively.^1,10^ TAVI has become the preferred treatment option in frail patients with severe AS.^11^ Similar to SAVR, the proportion of Heyde patients with gastrointestinal bleeding decreased significantly after TAVI in our study, albeit at a lower rate. Our results are consistent with the outcomes reported by Waldschmidt et al.^4^ The number of gastrointestinal bleeding episodes decreased in the months following TAVI in most patients with continuous bleeding (Figure 3). Late bleeding episodes (30–365 days), which others have recognized as an important cause of morbidity and mortality after TAVI, occurred in a minority (25%) of patients.^12^ Effects of TAVI on bleeding cessation were maintained up to 5 years thereafter in most (83%) patients (Figure S2).

We found that ≥mild PVL was associated with a higher risk of rebleeding episodes after TAVI (OR, 3.65). The presence of PVL maintains von Willebrand factor multimer deficiency, supporting the assumption that acquired von Willebrand disease plays a vital role in the pathogenesis.^18^ Indeed, balloon dilatation performed post-TAVI to reduce the degree of PVL resolves sustained acquired von Willebrand disease.^24^ Similarly, we reported fewer gastrointestinal bleeding episodes in patients who received a next-generation TAVI valve (1.1 versus 1.6), which could be a result of the lower rate of ≥mild PVL in this group (40% versus 60%). Since PVL is more prevalent after TAVI than SAVR, this could explain the difference in rates of bleeding cessation between both procedures.^25^ The design of procedural strategies to further reduce PVL after TAVI is pivotal.

Patients with gastrointestinal rebleeding more often used dual antiplatelet therapy or dual therapy with oral anticoagulants after TAVI than patients without rebleeding (93% versus 81%), but this difference was not significant (*P*=0.193). Previous studies reported contradicting results about the association between antithrombotic treatment strategies and gastrointestinal rebleeding.^1,4^ An explanation for these differing results could be that the bleeding diathesis caused by acquired von Willebrand disease outweighs that of antithrombotics. Moreover, as the recovery of von Willebrand factor halts angiogenesis, the influence of antithrombotics could lessen over time.^23^ Supporting this hypothesis, Dietrich et al^26^ recently reported that major late bleeding complications after TAVI were associated with unresolved acquired von Willebrand disease, while no relationship with the use of antithrombotics was found.

We also found that patients with rebleeding episodes more often had multiple angiodysplasias (52% versus 27%) located in the small bowel (30% versus 14%). These differences were not significant, likely due to the low number of patients, as only 14% underwent complete small bowel assessment. Notably, small bowel assessment is usually performed in patients with a more severe phenotype, which could contribute to the difference in rebleeding rates.^27^

Roughly 80% of patients with AS have acquired von Willebrand disease, but only a small fraction develops symptomatic angiodysplasias.^3,7^ We compared patients with Heyde syndrome to control patients who underwent TAVI and identified chronic kidney disease, hematologic disorders, and liver cirrhosis as additional risk factors for Heyde syndrome. These comorbidities are associated with the clinical severity risk of angiodysplasias.^17^ Interestingly, the risk of gastrointestinal rebleeding after TAVI was not related to any associated comorbidities, again underlining the importance of von Willebrand factor. Heyde patients also tended to have a smaller aortic valve area than control patients (0.71 versus 0.80 cm^2^), albeit not significant (*P*=0.069). Previous studies reported lower von Willebrand factor levels in patients with smaller aortic valve areas and found a significantly more reduced aortic valve area in patients with acquired von Willebrand disease (0.7 versus 0.8 cm^2^).^5,28^

We also found that patients with Heyde syndrome have a higher rate of periprocedural bleeding than control patients (adjusted OR, 2.55 [95% CI, 1.37–4.73]), which was mainly attributed to gastrointestinal bleeding. Angiodysplasia-related bleeding after aortic valve replacement can be massive and even fatal.^29,30^ The high rate of periprocedural gastrointestinal bleeding seems contradictory, as the overall number of bleeding episodes decreases after TAVI. Nevertheless, previous research has linked anemia before valve replacement to an increased risk of periprocedural bleeding.^31^ Even though the von Willebrand factor increases after valve replacement, it could take several days before normal levels are achieved.^32^ The use of heparin during the procedure, while the characteristic bleeding diathesis of Heyde syndrome is still present, could result in a surplus of bleeding. Since most patients with AS have acquired von Willebrand disease, desmopressin therapy has been applied successfully to increase von Willebrand factor multimers and reduce periprocedural bleeding.^30,33^

We did not find a significant difference in mortality rates between Heyde syndrome and control patients, and no deaths related to gastrointestinal bleeding were reported in our cohort.

Current treatment options for angiodysplasias are often inadequate, as lesions tend to be multiple and are often difficult to reach endoscopically.^3^ Our study highlights the efficacy of TAVI to treat angiodysplasias in Heyde syndrome. As indications of TAVI continue to expand, more patients with Heyde syndrome will be subjected to this intervention.^11^ It is important that clinicians recognize Heyde syndrome, particularly in patients with idiopathic anemia and associated comorbidities, and realize the benefits of TAVI, as the often frail patients could be denied the procedure due to presumed lack of improvement.^34^ Moreover, Heyde syndrome, consisting of all 3 associated disorders, has also been reported in mild AS.^35^ This raises the question whether patients with Heyde syndrome could benefit earlier on from TAVI, especially since the incidence of postprocedural PVL has dropped over the years.^36^ Some centers have already implemented valve replacement to treat severe gastrointestinal bleeding rather than AS symptoms. A small prospective study reported that SAVR also effectively reduces gastrointestinal bleeding in these patients.^37^

An evident strength of our study is that our cohort consisted of patients who were part of a prospective registry.^15^ The registry captured all AS patients necessitating TAVI since the introduction of the procedure in our center in 2008 and allowed us to have an extended follow-up. A limitation of this study is that small bowel assessment was not part of the clinical workup, therefore, underestimating the true prevalence of Heyde syndrome. We decided to also include patients with a high suspicion of angiodysplasias, as vascular malformations are the dominant cause of small bowel bleedings in elderly patients, particularly in those with acquired von Willebrand disease.^5,27^ Excluding these patients could falsely diminish TAVI effects, as patients with a more severe phenotype generally undergo additional small bowel assessment.^27^ To circumvent misclassification bias, we performed sensitivity analyses in patients with confirmed Heyde syndrome (44/70). As expected, patients with endoscopically established angiodysplasias had slightly more bleeding episodes in the year before TAVI (3.6 versus 3.2), but the decrease in bleeding episodes in the year after TAVI was identical.

In conclusion, patients with Heyde syndrome experience a durable reduction of gastrointestinal bleeding following TAVI, which becomes evident after the periprocedural period. Postprocedural PVL is associated with gastrointestinal bleeding recurrence.

## Article Information

### Sources of Funding

This study was funded by the Netherlands Organisation for Health Research and Development (ZonMw; grant number 848017006) and the Radboud University Medical Center. ZonMw and the Radboud University Medical Center had no role in study design, data collection, data analyses, data interpretation, or report preparation.

### Disclosures

Dr Drenth has received research funding from Gilead to support hepatitis C elimination in the Netherlands. Dr van Geenen has received research funding from Viatris, Boston Scientific, and Olympus and served as a consultant for MTW-Endoskopie and Microtech. Dr van Royen has received research funding from Abbott, Philips, and Biotronik; has served as a consultant for RainMed, Castor, and Medtronic; and received speaker fees from Abbott. The other authors report no conflicts.

### Supplemental Material

Tables S1–S4

Figures S1 and S2

## Supplementary Material



## References

[1] Thompson JL 3rd Schaff HV Dearani JA Park SJ Sundt TM 3rd Suri RM Blackshear JL Daly RC. Risk of recurrent gastrointestinal bleeding after aortic valve replacement in patients with Heyde syndrome. J Thorac Cardiovasc Surg. 2012;144:112–116. doi: 10.1016/j.jtcvs.2011.05.034 21864855 10.1016/j.jtcvs.2011.05.034

[2] Heyde EC. Gastrointestinal bleeding in aortic stenosis. N Engl J Med. 1958;259:196.

[3] García-Compeán D Del Cueto-Aguilera ÁN Jiménez-Rodríguez AR González-González JA Maldonado-Garza HJ. Diagnostic and therapeutic challenges of gastrointestinal angiodysplasias: a critical review and view points. World J Gastroenterol. 2019;25:2549–2564. doi: 10.3748/wjg.v25.i21.2549 31210709 10.3748/wjg.v25.i21.2549 PMC6558444

[4] Waldschmidt L Drolz A Heimburg P Goßling A Ludwig S Voigtländer L Linder M Schofer N Reichenspurner H Blankenberg S. Heyde syndrome: prevalence and outcomes in patients undergoing transcatheter aortic valve implantation. Clin Res Cardiol. 2021;110:1939–1946. doi: 10.1007/s00392-021-01905-z 34302190 10.1007/s00392-021-01905-z PMC8639542

[5] Tamura T Horiuchi H Imai M Tada T Shiomi H Kuroda M Nishimura S Takahashi Y Yoshikawa Y Tsujimura A. Unexpectedly high prevalence of acquired von Willebrand syndrome in patients with severe aortic stenosis as evaluated with a novel large multimer index. J Atheroscler Thromb. 2015;22:1115–1123. doi: 10.5551/jat.30809 26269004 10.5551/jat.30809

[6] George H Holcombe S Gupta R Croese J Tjahjadi C Walters D Raffel C Poon K Crowhurst J Clarke A. Do patients who are found to be anemic before transcatheter aortic valve implantation get worked up to determine potential sources of bleeding? J Gastroenterol Hepatol. 2017;32:39–40.27600190

[7] Goltstein LCMJ Grooteman KV Rocco A Holleran G Frago S Salgueiro PS Aparicio T Scaglione G Chetcuti Zammit S Prados-Manzano R. Effectiveness and predictors of response to somatostatin analogues in patients with gastrointestinal angiodysplasias: a systematic review and individual patient data meta-analysis. Lancet Gastroenterol Hepatol. 2021;6:922–932. doi: 10.1016/S2468-1253(21)00262-4 34508668 10.1016/S2468-1253(21)00262-4

[8] Vincentelli A Susen S Le Tourneau T Six I Fabre O Juthier F Bauters A Decoene C Goudemand J Prat A. Acquired von Willebrand syndrome in aortic stenosis. N Engl J Med. 2003;349:343–349. doi: 10.1056/NEJMoa022831 12878741 10.1056/NEJMoa022831

[9] Blackshear JL. Heyde syndrome: aortic stenosis and beyond. Clin Geriatr Med. 2019;35:369–379. doi: 10.1016/j.cger.2019.03.007 31230737 10.1016/j.cger.2019.03.007

[10] King RM Pluth JR Giuliani ER. The association of unexplained gastrointestinal bleeding with calcific aortic stenosis. Ann Thorac Surg. 1987;44:514–516. doi: 10.1016/s0003-4975(10)62112-1 3499881 10.1016/s0003-4975(10)62112-1

[11] Voigtländer L Seiffert M. Expanding TAVI to low and intermediate risk patients. Front Cardiovasc Med. 2018;5:92. doi: 10.3389/fcvm.2018.00092 30050909 10.3389/fcvm.2018.00092 PMC6052659

[12] Généreux P Cohen DJ Mack M Rodes-Cabau J Yadav M Xu K Parvataneni R Hahn R Kodali SK Webb JG. Incidence, predictors, and prognostic impact of late bleeding complications after transcatheter aortic valve replacement. J Am Coll Cardiol. 2014;64:2605–2615. doi: 10.1016/j.jacc.2014.08.052 25524339 10.1016/j.jacc.2014.08.052

[13] Godino C Lauretta L Pavon AG Mangieri A Viani G Chieffo A Galaverna S Latib A Montorfano M Cappelletti A. Heyde’s syndrome incidence and outcome in patients undergoing transcatheter aortic valve implantation. J Am Coll Cardiol. 2013;61:687–689. doi: 10.1016/j.jacc.2012.10.041 23391203 10.1016/j.jacc.2012.10.041

[14] von Elm E Altman DG Egger M Pocock SJ Gøtzsche PC Vandenbroucke JP; STROBE Initiative. The strengthening the reporting of observational studies in epidemiology (STROBE) statement: guidelines for reporting observational studies. Int J Surg. 2014;12:1495–1499. doi: 10.1016/j.ijsu.2014.07.01325046131

[15] Rooijakkers MJP van der Wulp K van Wely MH van Royen N. Transcatheter Aortic Valve Implantation Database of Radboud UMC. 2021. Dataset.

[16] van der Wulp K van Wely MH Schoon Y Vart P Olde Rikkert MGM Morshuis WJ van Royen N; Radboudumc TAVI Working Group. Geriatric assessment in the prediction of delirium and long-term survival after transcatheter aortic valve implantation. J Thorac Cardiovasc Surg. 2021;161:2095–2102.e3. doi: 10.1016/j.jtcvs.2020.02.076 32241615 10.1016/j.jtcvs.2020.02.076

[17] Grooteman KV Holleran G Matheeuwsen M van Geenen EJM McNamara D Drenth JPH. A risk assessment of factors for the presence of angiodysplasias during endoscopy and factors contributing to symptomatic bleeding and rebleeds. Dig Dis Sci. 2019;64:2923–2932. doi: 10.1007/s10620-019-05683-7 31190204 10.1007/s10620-019-05683-7 PMC6744377

[18] Kibler M Marchandot B Messas N Labreuche J Vincent F Grunebaum L Hoang VA Reydel A Crimizade U Kindo M. Primary hemostatic disorders and late major bleeding after transcatheter aortic valve replacement. J Am Coll Cardiol. 2018;72:2139–2148. doi: 10.1016/j.jacc.2018.08.2143 30360823 10.1016/j.jacc.2018.08.2143

[19] Blackshear JL Wysokinska EM Safford RE Thomas CS Shapiro BP Ung S Stark ME Parikh P Johns GS Chen D. Shear stress-associated acquired von Willebrand syndrome in patients with mitral regurgitation. J Thromb Haemost. 2014;12:1966–1974. doi: 10.1111/jth.12734 25251907 10.1111/jth.12734

[20] Ward SE O’Sullivan JM O’Donnell JS. The relationship between ABO blood group, von Willebrand factor, and primary hemostasis. Blood. 2020;136:2864–2874. doi: 10.1182/blood.2020005843 32785650 10.1182/blood.2020005843 PMC7751360

[21] Fay MP Proschan MA. Wilcoxon-Mann-Whitney or t-test? on assumptions for hypothesis tests and multiple interpretations of decision rules. Stat Surv. 2010;4:1–39. doi: 10.1214/09-SS051 20414472 10.1214/09-SS051 PMC2857732

[22] McHugh ML. The chi-square test of independence. Biochem Med (Zagreb). 2013;23:143–149. doi: 10.11613/bm.2013.018 23894860 10.11613/BM.2013.018 PMC3900058

[23] Randi AM Smith KE Castaman G. von Willebrand factor regulation of blood vessel formation. Blood. 2018;132:132–140. doi: 10.1182/blood-2018-01-769018 29866817 10.1182/blood-2018-01-769018 PMC6182264

[24] Van Belle E Rauch A Vincent F Robin E Kibler M Labreuche J Jeanpierre E Levade M Hurt C Rousse N. Von Willebrand factor multimers during transcatheter aortic-valve replacement. N Engl J Med. 2016;375:335–344. doi: 10.1056/NEJMoa1505643 27464202 10.1056/NEJMoa1505643

[25] Wagner G Steiner S Gartlehner G Arfsten H Wildner B Mayr H Moertl D. Comparison of transcatheter aortic valve implantation with other approaches to treat aortic valve stenosis: a systematic review and meta-analysis. Syst Rev. 2019;8:44. doi: 10.1186/s13643-019-0954-3 30722786 10.1186/s13643-019-0954-3 PMC6362570

[26] Dietrich L Kibler M Matsushita K Marchandot B Trimaille A Reydel A Diop B Truong PD Trung AM Trinh A. Impact of primary hemostasis disorders on late major bleeding events among anticoagulated atrial fibrillation patients treated by TAVR. J Clin Med. 2021;11:212. doi: 10.3390/jcm11010212 35011952 10.3390/jcm11010212 PMC8746148

[27] Zhang BL Chen CX Li YM. Capsule endoscopy examination identifies different leading causes of obscure gastrointestinal bleeding in patients of different ages. Turk J Gastroenterol. 2012;23:220–225. doi: 10.4318/tjg.2012.0338 22798110 10.4318/tjg.2012.0338

[28] Sedaghat A Kulka H Sinning JM Falkenberg N Driesen J Preisler B Hammerstingl C Nickenig G Pötzsch B Oldenburg J. Transcatheter aortic valve implantation leads to a restoration of von Willebrand factor (VWF) abnormalities in patients with severe aortic stenosis - incidence and relevance of clinical and subclinical VWF dysfunction in patients undergoing transfemoral TAVI. Thromb Res. 2017;151:23–28. doi: 10.1016/j.thromres.2016.12.027 28088607 10.1016/j.thromres.2016.12.027

[29] Varma P Misra M Radhakrishnan VV Neelakandhan KS. Fatal post-operative gastro intestinal hemorrhage because of angio-dysplasia of small intestine in aortic regurgitation. Interact Cardiovasc Thorac Surg. 2004;3:118–120. doi: 10.1016/S1569-9293(03)00233-0 17670193 10.1016/S1569-9293(03)00233-0

[30] Mahboobi SK. Heyde’s syndrome and postoperative bleeding after aortic valve replacement - Is there a role for prophylactic desmopressin? J Clin Anesth. 2019;56:142. doi: 10.1016/j.jclinane.2019.02.002 30785003 10.1016/j.jclinane.2019.02.002

[31] Nagao K Taniguchi T Morimoto T Shiomi H Ando K Kanamori N Murata K Kitai T Kawase Y Izumi C; CURRENT AS Registry Investigators. Anemia in patients with severe aortic stenosis. Sci Rep. 2019;9:1924. doi: 10.1038/s41598-018-36066-z30760807

[32] Pawlitschek F Keyl C Zieger B Budde U Beyersdorf F Neumann FJ Stratz C Nührenberg TG Trenk D. Alteration of von Willebrand factor after transcatheter aortic valve replacement in the absence of paravalvular regurgitation. Thromb Haemost. 2018;118:103–111. doi: 10.1160/17-07-0506 29304530 10.1160/17-07-0506

[33] Steinlechner B Zeidler P Base E Birkenberg B Ankersmit HJ Spannagl M Quehenberger P Hiesmayr M Jilma B. Patients with severe aortic valve stenosis and impaired platelet function benefit from preoperative desmopressin infusion. Ann Thorac Surg. 2011;91:1420–1426. doi: 10.1016/j.athoracsur.2011.01.052 21439546 10.1016/j.athoracsur.2011.01.052

[34] Kim DH Afilalo J Shi SM Popma JJ Khabbaz KR Laham RJ Grodstein F Guibone K Lux E Lipsitz LA. Evaluation of changes in functional status in the year after aortic valve replacement. JAMA Intern Med. 2019;179:383–391. doi: 10.1001/jamainternmed.2018.6738 30715097 10.1001/jamainternmed.2018.6738 PMC6439710

[35] Schwaiger JP Ludwiczek O Graziadei I Grander W. A vicious circle: Heyde syndrome in mild aortic stenosis. CASE (Phila). 2019;3:171–176. doi: 10.1016/j.case.2019.04.005 31468021 10.1016/j.case.2019.04.005 PMC6710854

[36] Winter MP Bartko P Hofer F Zbiral M Burger A Ghanim B Kastner J Lang IM Mascherbauer J Hengstenberg C. Evolution of outcome and complications in TAVR: a meta-analysis of observational and randomized studies. Sci Rep. 2020;10:15568. doi: 10.1038/s41598-020-72453-1 32968104 10.1038/s41598-020-72453-1 PMC7511292

[37] Rosa VEE Ribeiro HB Fernandes JRC Santis A Spina GS Paixão MR Pires LJT Bettega M Accorsi TAD Sampaio RO. Heyde’s syndrome: therapeutic strategies and long-term follow-up. Arq Bras Cardiol. 2021;117:512–517. doi: 10.36660/abc.20200371 34231795 10.36660/abc.20200371 PMC8462952

