# Supplementary material for: Reduction of Gastrointestinal Bleeding in Patients With Heyde Syndrome Undergoing Transcatheter Aortic Valve Implantation
Source: Circ Cardiovasc Interv. 2022 Jul 5;15(7):e011848. doi: 10.1161/CIRCINTERVENTIONS.122.011848 (PMC9287099; doi:10.1161/CIRCINTERVENTIONS.122.011848)
Supplement: Supplementary file 1 [file hcv-15-e011848-s001.pdf]

SUPPLEMENTAL MATERIAL

Reduction of Gastrointestinal Bleeding in Patients with Heyde syndrome undergoing Transcatheter Aortic Valve Implantation

Lia C.M.J. Goltstein, Maxim J.P. Rooijakkers, Natasha C.C. Görtjes, Reinier P. Akkermans, Erwin S. Zegers, Ron Pisters, Marleen H. van Wely, Kees van der Wulp, Joost P.H. Drenth, Erwin J.M. van Geenen, Niels van Royen.

Figure S1. Cumulative incidence of gastrointestinal bleeding up to 1-year after TAVI........... 1

Figure S2. Cumulative incidence of gastrointestinal bleeding up to 5-years after TAVI 2

Table S1. Inclusion and exclusion criteria of Heyde and control patients 3

Table S2. Adjusted Bleeding Academic Research Consortium classification 4

Table S3. Differences in bleeding episodes and corresponding healthcare 1-year before and after TAVI in patients with confirmed angiodysplasias (N=44).. 5

Table S4. Differences in bleeding episodes and corresponding healthcare 1-year before and after TAVI in patients who received a next-generation valve (N=45)...................................... 6

Figure S1. Cumulative incidence of gastrointestinal bleeding up to 1-year after TAVI

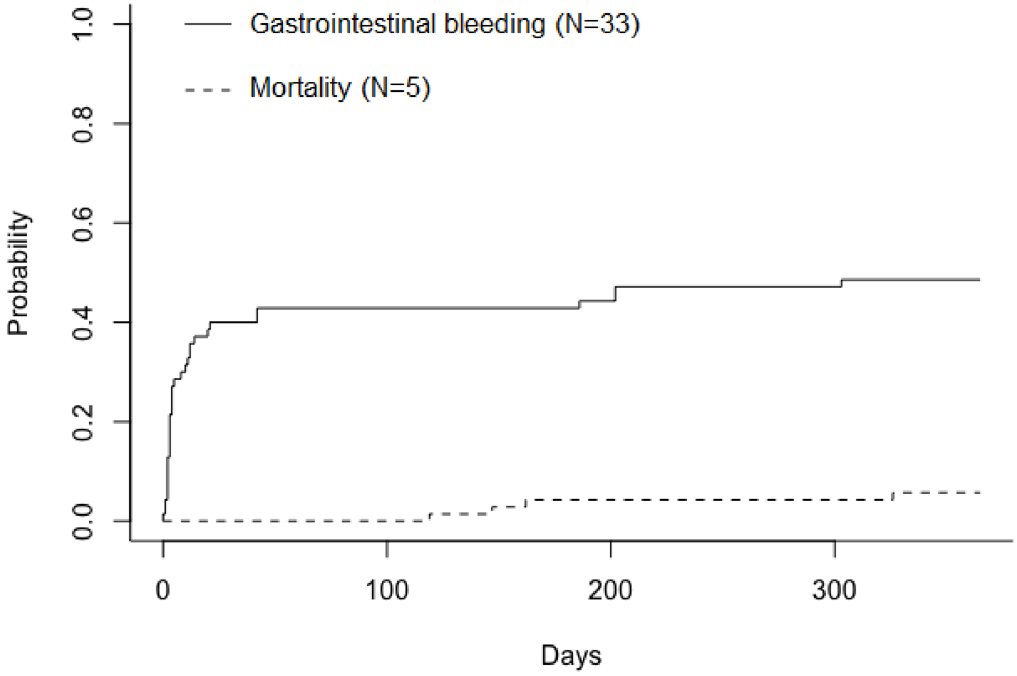

Figure S2. Cumulative incidence of gastrointestinal bleeding up to 5-years after TAVI

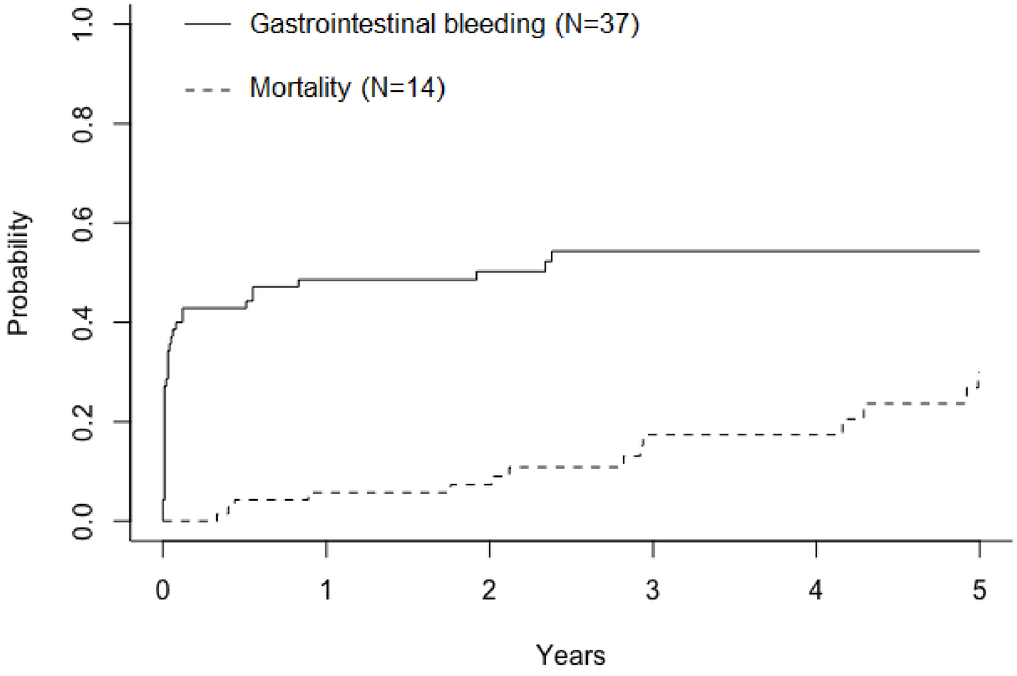

Table S1. Inclusion and exclusion criteria of Heyde and control patients

Heyde patients	Control patients
**Inclusion criteria**	
Age ≥ 18 years	Age ≥ 18 years
Underwent TAVI procedure in Radboud University Medical Center between December 2008 and June 2020	Underwent TAVI procedure in Radboud University Medical Center between December 2008 and June 2020
Severe AS defined as:<br>• AVA < 1 cm^2 AND/OR;<br>• PGmean > 40 mmHg AND/OR;<br>• Vmax across aortic valve > 4 m/s.	Severe AS defined as:<br>• AVA < 1 cm^2 AND/OR;<br>• PGmean > 40 mmHg AND/OR;<br>• Vmax across aortic valve > 4 m/s.
Overt or occult (IDA or positive FOBT) gastrointestinal bleeding episodes with (high suspicion of) angiodysplasias, defined as:<br>• Endoscopic diagnosis of angiodysplasias OR<br>• Unknown etiology after prior gastroscopy and colonoscopy, but without small bowel assessment	
**Exclusion criteria**	
Patients did not give consent to review data	Patients did not give consent to review data
No available data from referring center on gastrointestinal bleeding episodes 1 year before and 1 year after TAVI	Gastrointestinal bleeding episodes in medical history in which (concomitant) presence of angiodysplasias could not be ruled out because of incomplete endoscopic evaluation (consisting of prior gastroscopy, colonoscopy, and complete small bowel assessment)
Endoscopic diagnosis other than angiodysplasias in medical history or reported up to one year after TAVI likely to contribute to gastrointestinal bleeding (e.g., ulcer or cancer)	

AVA=Aortic valve area, EGD=Esophagogastroduodenoscopy, FOBT=Fecal occult blood test, IDA=Iron deficiency anemia, PG= peak gradient, TAVI=Transcatheter aortic valve implantation, Vmax=Peak aortic valve velocity.

Table S2. Adjusted Bleeding Academic Research Consortium classification

**Type 2**	Any overt or occult, actionable sign of hemorrhage that does not fit the criteria for type 3, 4, or 5, but does meet at least one of the following criteria:<br>1) Requiring nonsurgical, medical intervention by a health care professional,<br>2) Leading to hospitalization or increased level of care; or<br>3) Prompting evaluation*
**Type 3**	
• **Type 3A**	Overt or occult bleeding plus hemoglobin drop of 3 to < 5 g/dL (provided hemoglobin drop is related to bleed). Any transfusion with overt or occult bleeding
• **Type 3B**	Overt or occult bleeding plus hemoglobin drop of 5 or > g/dL (related to bleed), Cardiac tamponade, bleeding requiring surgical intervention for control, bleeding requiring intravenous vasoactive agents†
**Type 4**	Transfusion of 5 or > whole blood or packed cells within 48 hours
**Type 5**	Fatal bleeding

*A positive fecal occult blood test and endoscopic evaluation without application of treatment modalities were not scored. †Endoscopic treatment is also considered to be a surgical intervention.

Table S3. Differences in bleeding episodes and corresponding healthcare 1-year before and after TAVI in patients with confirmed angiodysplasias (N=44)

	**Mean ± 95% CI before TAVI**	**Mean ± 95% CI after TAVI**		***P*-value**
Hemoglobin level (g/dL)	10.3 (9.8–11.0)	11.1 (10.6–11.8)		**0.041**
Ferritin level	49 (13 to 85)	92 (56 to 128)		0.082
	**Geometric mean ± 95% CI**	**Geometric mean ± 95% CI**	**Incidence Rate Ratio [IRR]**	***P*-value**
Bleeding episodes	3.6 (2.6–5.0)	2.0 (1.4–2.9)	0.6 (0.4–0.9)	**0.021**
• BARC Type II	0.1 (0.0–0.2)	0.0 (0.0–0.2)	0.4 (0.0–3.6)	0.384
• BARC Type IIIa	2.8 (2.0–4.1)	1.7 (1.1–2.6)	0.6 (0.4–1.0)	0.072
• BARC Type IIIb	0.7 (0.5–1.0)	0.4 (0.2–0.7)	0.6 (0.3–2.0)	0.102
• BARC Type IV	0.5 (0.3–0.9)	0.4 (0.2–0.8)	0.8 (0.4–1.8)	0.615
Blood transfusions	5.7 (3.6–9.1)	3.6 (2.2–5.8)	0.6 (0.3–1.2)	0.162
Iron infusions	0.9 (0.6–1.5)	0.5 (0.3–1.0)	0.6 (0.3–1.2)	0.144
Day-care treatment	2.0 (1.3–2.9)	1.7 (1.1–2.6)	0.9 (0.5–1.5)	0.602
Emergency department visits	1.0 (0.6–1.7)	0.9 (0.5–1.6)	0.9 (0.4–2.0)	0.837
Hospital admissions (days)	7.5 (3.4–16.3)	4.2 (1.9–9.3)	0.6 (0.2–1.7)	0.306
Endoscopic procedures	1.7 (1.1–2.5)	1.2 (0.8–1.8)	0.7 (0.4–1.3)	0.304

Values represent the mean or the IRR interaction (95% confidence intervals [95% CI] in the column). BARC=Bleeding Academic Research Consortium, IRR=Incidence Rate Ratio.

Table S4. Differences in bleeding episodes and corresponding healthcare 1-year before and after TAVI in patients who received a next-generation valve (N=45)

	**Mean ± 95% CI before TAVI**	**Mean ± 95% CI after TAVI**		***P*-value**
Hemoglobin level (g/dL)	11.3 (7.9–14.7)	12.1 (8.6–15.7)		0.084
Ferritin level	66 (39 to 93)	76 (48 to 103)		0.591
	**Geometric mean ± 95% CI**	**Geometric mean ± 95% CI**	**Incidence Rate Ratio [IRR]**	***P*-value**
Bleeding episodes	2.5 (1.7–3.7)	1.1 (0.7–1.8)	0.5 (0.3–0.8)	**0.007**
• BARC Type II	0.0 (0.0–0.2)	0.0 (0.0–0.0)	0.0 (0.0–6.0)	0.577
• BARC Type IIIa	2.6 (1.7–3.8)	1.6 (1.1–2.5)	0.6 (0.4–1.2)	0.135
• BARC Type IIIb	0.6 (0.4–1.1)	0.6 (0.3–1.1)	1.0 (0.4–2.2)	0.925
Blood transfusions	5.7 (3.6–9.1)	3.6 (2.2–5.8)	0.8 (0.4–1.6)	0.456
Iron infusions	1.1 (0.7–1.7)	0.5 (0.3–0.9)	0.5 (0.2–1.0)	0.062
Day-care treatment	1.7 (1.0–2.6)	1.6 (1.0–2.5)	0.9 (0.5–1.8)	0.840
Emergency department visits	1.0 (0.5–1.7)	0.7 (0.4–1.3)	0.8 (0.3–1.7)	0.505
Hospital admissions (days)	6.0 (2.7–13.6)	2.4 (1.1–5.6)	0.4 (0.1–1.3)	0.123
Endoscopic procedures	1.2 (0.7–1.9)	1.1 (0.7–1.8)	0.9 (0.5–1.8)	0.921

Values represent the mean or the IRR interaction (95% confidence intervals [95% CI] in the column). BARC=Bleeding Academic Research Consortium, IRR=Incidence Rate Ratio.
